# Theoretical Analysis of GeSn Quantum Dots for Photodetection Applications

**DOI:** 10.3390/s24041263

**Published:** 2024-02-16

**Authors:** Pin-Hao Lin, Soumava Ghosh, Guo-En Chang

**Affiliations:** Department of Mechanical Engineering, and Advanced Institute of Manufacturing with High-Tech Innovations (AIM-HI), National Chung Cheng University, Chiayi 621301, Taiwan; arron@alum.ccu.edu.tw (P.-H.L.); sghosh@ccu.edu.tw (S.G.)

**Keywords:** GeSn alloy, quantum dots, photodetectors, image sensing

## Abstract

GeSn alloys have recently emerged as complementary metal–oxide–semiconductor (CMOS)-compatible materials for optoelectronic applications. Although various photonic devices based on GeSn thin films have been developed, low-dimensional GeSn quantum structures with improved efficiencies hold great promise for optoelectronic applications. This study theoretically analyses Ge-capped GeSn pyramid quantum dots (QDs) on Ge substrates to explore their potential for such applications. Theoretical models are presented to calculate the effects of the Sn content and the sizes of the GeSn QDs on the strain distributions caused by lattice mismatch, the band structures, transition energies, wavefunctions of confined electrons and holes, and transition probabilities. The bandgap energies of the GeSn QDs decrease with the increasing Sn content, leading to higher band offsets and improved carrier confinement, in addition to electron–hole wavefunction overlap. The GeSn QDs on the Ge substrate provide crucial type–I alignment, but with a limited band offset, thereby decreasing carrier confinement. However, the GeSn QDs on the Ge substrate show a direct bandgap at higher Sn compositions and exhibit a ground-state transition energy of ~0.8 eV, rendering this system suitable for applications in the telecommunication window (1550 nm). These results provide important insights into the practical feasibility of GeSn QD systems for optoelectronic applications.

## 1. Introduction

Although group-III–V- and II–VI-based photodetectors (PDs) show very promising performance in modern optical communication [1,2,3], their incompatibility with Si-based complementary metal–oxide–semiconductor (CMOS) processing technology makes them bulky and expensive. In contrast, group-IV Si and Ge PDs are compatible with CMOS technology, but their lower cut-off wavelengths of ~1.1 and 1.5 µm, respectively, limit their applications in the short-wave infrared (SWIR) [1.4–3 µm] and mid-infrared (MIR) [3–8 µm] regions [4]. This bottleneck has been circumvented by growing high-quality GeSn alloys on Si substrates bearing a suitable buffer layer using molecular beam epitaxy (MBE) or chemical vapour deposition (CVD) [5,6,7]. Meanwhile, the addition of Sn to Ge has been demonstrated to dramatically modulate the band structure and reduce Δ*E*_ΓL_, eventually transforming the bandgap of GeSn from indirect to direct at a Sn content of >6.6% and enabling efficient direct-gap transitions [8]. Moreover, GeSn alloys show bandgap tunability, a high carrier saturation velocity [9], high carrier mobility [10], and a large absorption coefficient [11]. These unique characteristics have encouraged the development of efficient GeSn-based SWIR and MIR PDs [8,9,10,11,12,13,14,15,16,17]. Furthermore, a special momentum (k)-space carrier separation scheme enhances the optical performance of the GeSn PDs [18].

However, GeSn PDs suffer from a large dark current that restricts their practical applications [19]. Thermally generated carriers are one of the sources of this dark current, and external cooling is currently the best solution to overcome this problem [20]. However, this approach makes PD-based IR cameras bulky, heavy, and expensive. On the other hand, to extend the photodetection range, a large Sn concentration is required, which also increases the dark current, and the solid solubility of Sn in Ge is only ~1–2% under thermal equilibrium conditions [21,22]. Although low-temperature growth methods have proven effective in breaking this limit by introducing greater amounts of Sn into GeSn alloys, these methods also generate defects, thus significantly degrading the material. As a result, Shockley–Read–Hall (SRH) nonradiative recombination increasingly occurs, thereby increasing the dark current [19]. In addition, higher Sn contents have been found to substantially increase the lattice mismatch between GeSn alloys and the Si substrate, which reduces the critical thickness [23]. When the thickness of the GeSn layer exceeds this critical thickness, strain relaxation leads to considerable defect formation, which is even more pronounced at a higher Sn content. Although using a composition-graded GeSn buffer layer can suppress defect generation, the top GeSn active layer is far from the Si substrate, ultimately causing a loss in the optical confinement, thus reducing the optical responsivity.

One promising approach for overcoming the aforementioned material challenges is to develop a GeSn quantum dot (QD) structure. The use of QDs achieves high IR absorption [24], a low dark current [25], high detectivity [26], multi-spectral response [27], and good IR imaging capabilities [28,29]. Furthermore, owing to the zero-dimensional structure of QDs, QD-based IR PDs can even maintain a low dark current when operated at high temperatures. Therefore, QD IR PDs have been attracting the interest of many researchers. When a sufficient lattice mismatch exists between the epitaxial material and the substrate, QDs can be grown in the Stranski–Krastanow (SK) growth mode. In this context, SK-mode QDs based on highly lattice-matched systems, including Ge QDs on Si [30,31,32] and silicon-on-insulator (SOI) systems [33,34], have been widely investigated. This approach has been found to alleviate the material growth challenges associated with GeSn alloys. Furthermore, various reports have described the significant strain that is induced in SK-QDs, which can significantly modify their band structures. Although only a few theoretical studies on GeSn QDs have been reported thus far [35,36,37], they have demonstrated a promising response for GeSn QDs both in a colloidal structure [35] and a CMOS-compatible structure [36,37]. However, those works mainly focused on their application as light emitters. It is therefore of interest to study the fundamental properties of GeSn QDs to determine their potential for use in detection and image sensing applications, which may reduce the cost of IR cameras.

To that end, we herein present a comprehensive theoretical analysis of the optoelectronic properties of GeSn QDs. Initially, the strain distribution of GeSn QDs grown on Ge substrates capped by Ge is calculated, and subsequently, the electronic band structures and the confined energies and wavefunctions of the electrons and holes are determined. The results are anticipated to provide insights into the optoelectronic properties of GeSn QD systems for their future incorporation into optoelectronic and image sensing devices.

## 2. Methods

### 2.1. GeSn Quantum Dot (QD) Structure

**Figure 1:**
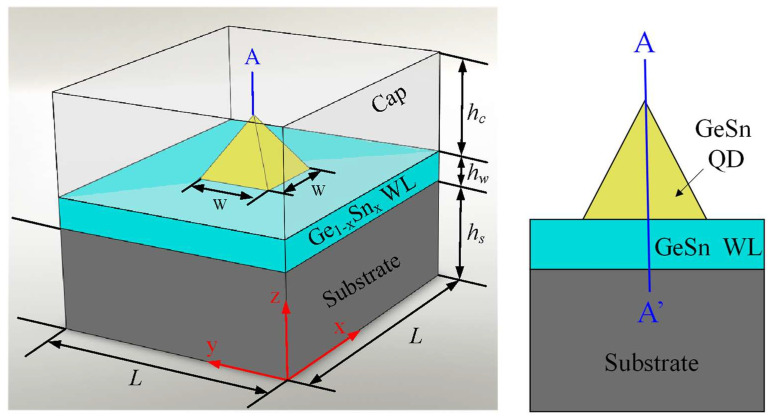
Schematic diagram of a GeSn QD.

Figure 1 shows the modelled GeSn QD structure, consisting of a (001)-oriented Ge substrate, a Ge_1−x_Sn_x_ wetting layer (WL), a pyramid-shaped Ge_1−x_Sn_x_ QD, and a cap layer. The width and height of the pyramid-shaped Ge_1−x_Sn_x_ QDs were set to *w* = 12 nm and *h* = 6 nm, respectively (i.e., *w* = 2*h*), while the thicknesses of the wetting and cap layers were *h*_w_ = 0.5 nm and *h*_c_ = 15 nm, respectively. To obtain a high-quality GeSn/Ge QD system, highly controllable methods [36,37] can maintain a high Sn content and the uniformity of the GeSn QDs, making them credible for photodetection applications.

### 2.2. Theoretical Models

#### 2.2.1. Strain Analysis

The strain fields in the Ge_1−x_Sn_x_ QDs were induced by the lattice mismatch between the Ge_1−x_Sn_x_ layer and substrate, as outlined in Equation (1):

(1) $$\alpha =\frac{a\left(sub\right)-a\left({Ge}_{1-x}{Sn}_{x}\right)}{a\left({Ge}_{1-x}{Sn}_{x}\right)}$$

where $a\left(sub\right)$ and $a\left({Ge}_{1-x}{Sn}_{x}\right)$ represent the lattice constants of the substrate and Ge_1−x_Sn_x_, respectively. The lattice constant of Ge_1−x_Sn_x_ can be calculated using Equation (2) [9]:

(2) $$a\left({Ge}_{1-x}{Sn}_{x}\right)=\left(1-x\right)a_{Ge}+xa_{Sn}+x(1-x)\theta_{GeSn}$$

where $a_{Ge}$ and $a_{Sn}$ denote the lattice constants of Ge and Sn, respectively, and $\theta_{GeSn}$ = 0.041 [9] is the bowing parameter. This lattice mismatch served as the initial strain and induced further elastic deformation in the entire structure. The induced strain fields were numerically simulated using a previous method [38]. All the parameters related to GeSn used in this study were deduced by the linear interpolation method from the characteristics of Ge and Sn obtained in previous studies [11,39].

#### 2.2.2. Band Structures

The various bands present in each structure were determined using the model solid theory considering the strain effect [40]. In the absence of strain, the valence band (VB) edge can be calculated using Equation (3) [40,41]:

(3) $$E_{V}^{0}=E_{V,av}+\frac{∆}{3}$$

where $E_{V,av}$ denotes the average position of the VB and Δ is the spin–orbit splitting energy. The unstrained conduction band (CB) minima can then be evaluated as follows [40,41]:

(4) $$E_{C,\eta }^{0}=E_{V,av}+\frac{∆}{3}+E_{g}$$

where $E_{g}$ is the unstrained bandgap. In addition, the unstrained bandgap energy of GeSn at *T* = 300 K can be described by Equation (5):

(5) $$E_{g}\left({Ge}_{1-x}{Sn}_{x}\right)=\left(1-x\right)E_{g}\left(Ge\right)+xE_{g}\left(Sn\right)+x\left(1-x\right)b_{w}$$

where $E_{g}\left(Ge\right)$ and $E_{g}\left(Sn\right)$ are the bandgap energies of Ge and Sn, respectively, and $b_{w}$ *=* 2.42 and 0.89 eV represent the bowing parameters for the Γ- and L-valleys, respectively [8]. The bandgap energies and other parameters of Ge and Sn are listed in Table 1.

The CB and VB offsets in the absence of strain can be expressed as follows:

(6) $$\Delta E_{C}^{0}=\left|{\left(E_{C}^{0}\right)}_{GeSn}-{\left(E_{C}^{0}\right)}_{Sub}\right|$$

(7) $$\Delta E_{V}^{0}=\left|{\left(E_{V}^{0}\right)}_{GeSn}-{\left(E_{V}^{0}\right)}_{Sub}\right|$$

Under strained conditions, the CB minima and the heavy hole (HH) and light hole (LH) maxima in the VB of GeSn can be expressed as follows [40,41]:

(8a) $$E_{C,\Gamma }\left({Ge}_{1-x}{Sn}_{x}\right)=\Delta E_{C}^{0}+a_{C}\left(\varepsilon_{xx}+\varepsilon_{yy}+\varepsilon_{zz}\right)$$

(8b) $$E_{C,L}\left({Ge}_{1-x}{Sn}_{x}\right)=\Delta E_{C,L}^{0}+a_{C}\left(\varepsilon_{xx}+\varepsilon_{yy}+\varepsilon_{zz}\right)$$

(8c) $$E_{HH}\left({Ge}_{1-x}{Sn}_{x}\right)=\Delta E_{V}^{0}+a_{V}\left(\varepsilon_{xx}+\varepsilon_{yy}+\varepsilon_{zz}\right)-b\left[\varepsilon_{zz}-\frac{1}{2}\left(\varepsilon_{xx}+\varepsilon_{yy}\right)\right]$$

(8d) $$E_{LH}\left({Ge}_{1-x}{Sn}_{x}\right)=\Delta E_{V}^{0}+a_{V}\left(\varepsilon_{xx}+\varepsilon_{yy}+\varepsilon_{zz}\right)+b\left[\varepsilon_{zz}-\frac{1}{2}\left(\varepsilon_{xx}+\varepsilon_{yy}\right)\right]$$

In contrast, the CB minima and the HH and LH maxima of the substrate can be calculated as follows [40,41]:

(9a) $$E_{C,\Gamma }\left(sub\right)=a_{C}\left(\varepsilon_{xx}+\varepsilon_{yy}+\varepsilon_{zz}\right)$$

(9b) $$E_{C,L}\left(sub\right)={\Delta E_{C,L}^{0}+a}_{C}\left(\varepsilon_{xx}+\varepsilon_{yy}+\varepsilon_{zz}\right)$$

(9c) $$E_{HH}\left(sub\right)=a_{V}\left(\varepsilon_{xx}+\varepsilon_{yy}+\varepsilon_{zz}\right)-b\left[\varepsilon_{zz}-\frac{1}{2}\left(\varepsilon_{xx}+\varepsilon_{yy}\right)\right]$$

(9d) $$E_{LH}\left(sub\right)=a_{V}\left(\varepsilon_{xx}+\varepsilon_{yy}+\varepsilon_{zz}\right)+b\left[\varepsilon_{zz}-\frac{1}{2}\left(\varepsilon_{xx}+\varepsilon_{yy}\right)\right]$$

where $a_{C}$ and $a_{V}$ represent the hydrostatic deformation potentials of the CB and VB, respectively, and b denotes the shear deformation potential of the VB. From Equations (8a–d) and (9a–d) we can calculate the direct and indirect bandgap of the active layer and substrate under strained conditions.

#### 2.2.3. Quantum Confined States

Based on the Schrödinger equation, the confined states and inter-band transition energies of the carriers can be calculated by Equation (10) [42]:

(10) $$-\frac{\hbar^{2}}{2}\nabla \left(\frac{1}{m^{*}\left(\vec{r}\right)}\nabla \Psi \left(\vec{r}\right)\right)+V\left(\vec{r}\right)\Psi \left(\vec{r}\right)=E\Psi \left(\vec{r}\right)$$

where ℏ is the reduced Planck constant, $m^{*}$ is the effective mass, $\vec{r}$ is the three-dimensional coordinate vector, Ψ represents the carrier wave function, E denotes the carrier energy, and V is the confinement potential of the carrier obtained from the band discontinuity.

#### 2.2.4. Optical Absorption Coefficient

The optical absorption coefficient of the Ge_1−x_Sn_x_ QDs can be calculated using Fermi’s golden rule with a Lorentzian line shape [40]:

(11) $$\alpha \left(\hbar \omega \right)=\frac{\pi e^{2}}{n_{r}c\varepsilon_{0}m_{0}^{2}\omega }\times \frac{1}{V_{0}}\sum_{\sigma }{\left|\hat{e}.p_{cv}\right|}^{2}\times I_{ab}\times \frac{\left(\frac{\Gamma }{2\pi }\right)}{{\left(E_{a}-E_{b}-\hbar \omega \right)}^{2}+{\left(\Gamma /2\right)}^{2}}$$

where e is the elementary charge, $n_{r}$ is the background refractive index, $\varepsilon_{0}$ is the permittivity, $m_{0}$ is the free electron mass, $V_{0}$ is the volume of the structure, $E_{a}$ and $E_{b}$ are the energy levels in the CB and VB, respectively, $\left|\hat{e}.p_{cv}\right|$ is the momentum matrix, and $I_{ab}$ is the electron–hole wavefunction overlap. Finally, Γ is the full-width-at-half-maximum of the Lorentzian shape, which was Γ = 20 meV in our calculations. For device applications, we consider normal incident photodetectors, where only transverse electric (TE) polarized light is absorbed by the material. Thus, the squared moment matrix for TE polarization is equal to $3M_{b}^{2}/2$ $\left(M_{b}^{2}/2\right)$ for the optical transition from HH (LH) sub-bands to Γ sub-bands, respectively.

## 3. Results

**Figure 2:**
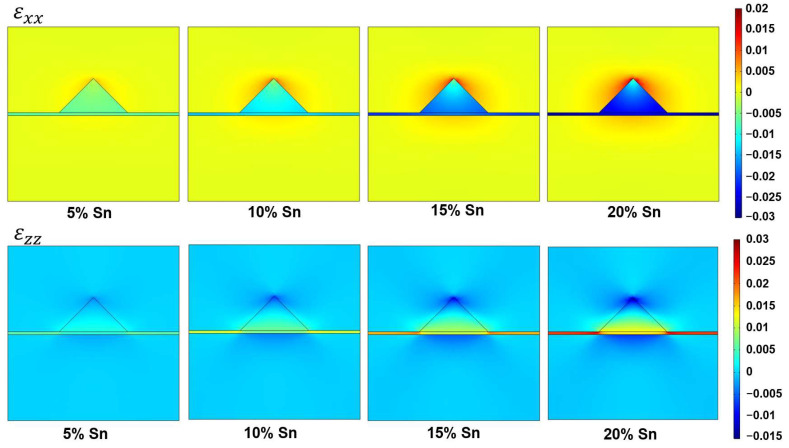
Strain distributions along the AA′ axis for the Ge/GeSn/Ge QDs containing different Sn contents (*w* = 12 nm, *h* = 6 nm).

By using the finite element method (FEM) in COMSOL Multiphysics, we analysed the strain, bandgap, and carrier confinement inside the GeSn QDs. Figure 2 shows the different strain distributions for the Ge/GeSn/Ge QD system with Sn contents ranging from 5% to 20%. At x = 5%, the small lattice mismatch of ~0.7% induces an extremely small compressive strain. Upon increasing the Sn content, the lattice mismatch increases, generating a higher strain. However, even at a Sn content of 20%, only a 2.97% lattice mismatch occurs. This can be accounted for by considering that inside the GeSn QDs, $\varepsilon_{xx}$ is negative because of the lattice mismatch. Thus, when moving from the Ge substrate to the Ge cap layer, the magnitude of the strain decreases. In contrast, $\varepsilon_{zz}$ is positive owing to the Poisson effect.

**Figure 3:**
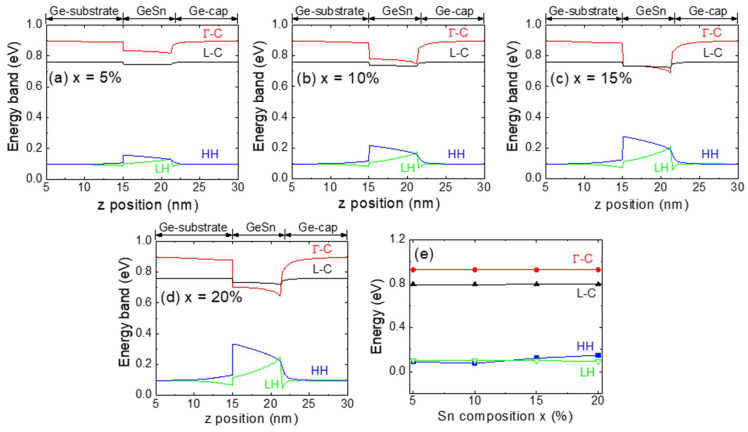
Calculated electronic band structures for the Ge/GeSn/Ge QD systems along their AA′ axes for different Sn contents: (**a**) x = 5%, (**b**) x = 10%, (**c**) x = 15%, and (**d**) x = 20% (*w* = 12 nm, *h* = 6 nm). (**e**) Calculated energy band variation upon altering the Sn content in the Ge_1−x_Sn_x_ layer.

Figure 3a–d illustrates the electronic band structures of the GeSn QDs (*w* = 12 nm, *h* = 6 nm) with various Sn contents on the Ge substrate along their AA′ axes. The Ge/GeSn/Ge interface creates a type-I band alignment. In addition, upon increasing the Sn content, both the Γ-CB and L-CB in the GeSn QDs moves downwards. Furthermore, the Γ-CB shifts downwards more rapidly because of the negative direct bandgap of Sn [8] and crosses the L-CB at higher Sn contents (i.e., >10%), indicating a direct bandgap. Moreover, increasing the Sn content is found to increase the compressive strain that lifts the HH over the LH. Additionally, at a lower Sn content, the Ge/GeSn/Ge system provides a lower degree of strong carrier confinement in the higher-energy state owing to the lower band offset. However, an increase in Sn content reduces the direct bandgap of GeSn, which increases the direct band offset, thereby enhancing carrier confinement. Figure 3e shows the band energy variations obtained for the QDs containing various Sn contents, and clearly demonstrates that upon increasing the Sn content, the Γ-CB and L-CB move downwards, as do the HH and LH bands.

**Figure 4:**
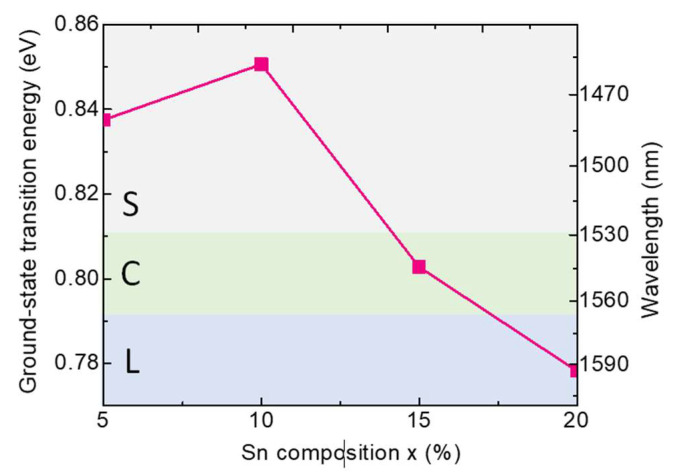
Calculated ground-state transition energies for the Ge/GeSn/Ge QD systems containing different Sn contents (*w* = 12 nm, *h* = 6 nm).

Figure 4 shows the ground-state transition energy as a function of the Sn content for the Ge/GeSn/Ge QD system, which initially increases slightly owing to the downward movement of the HH band. However, beyond a Sn content of 10%, the ground-state transition energy decreases because of the reduction in the effective bandgap. Another important feature of this figure is the fact that the effective bandgap covers the L-band of the optical communication window. In addition, the direct bandgap determined for this system falls within the recently developed telecommunication window of 1550 nm, thereby indicating the suitability of the Ge/GeSn/Ge QD system for telecommunication applications.

**Figure 5:**
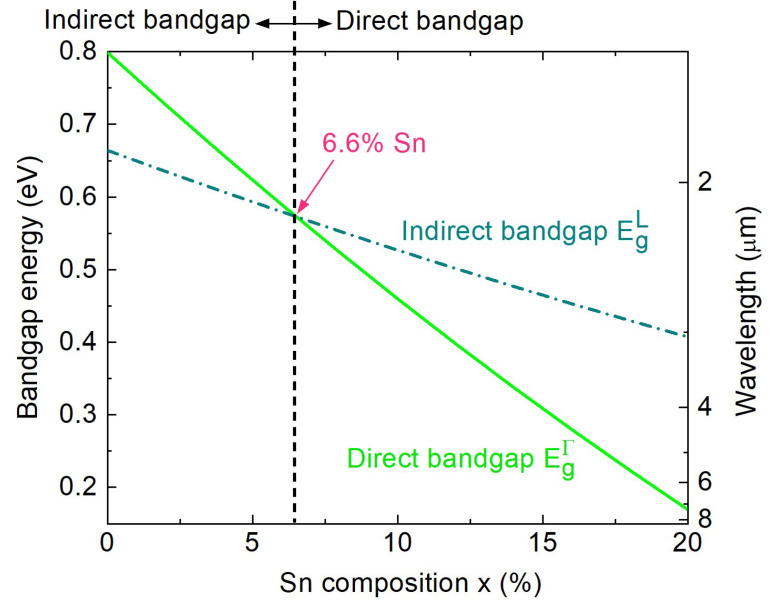
Calculated bandgaps of the GeSn alloy as a function of the Sn composition.

To better understand the impact of Sn alloying on the direct and indirect bandgaps, we calculated these bandgaps as functions of the Sn composition using Equation (5), and the results are plotted in Figure 5. Evidently, an increase in the Sn content reduces both the direct and indirect bandgaps of the alloy. However, owing to the negative direct bandgap of Sn, the Γ-valley moves more rapidly than the L-valley and finally crosses it at ~6.6% Sn. Therefore, at higher Sn compositions, the GeSn alloy has a direct bandgap. This theoretical finding is in good agreement with studies that considered band mixing effects in GeSn alloys [43].

**Figure 6:**
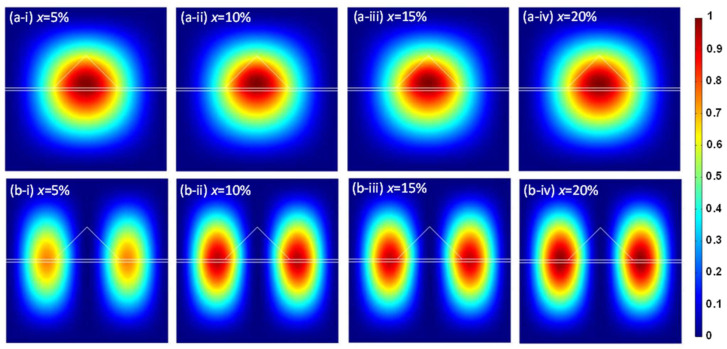
Electron probability densities for (**a**) the ground state and (**b**) the first excited state in the Γ-CB for Ge/GeSn/Ge QD systems with different Sn contents: (**a-i**,**b-i**) x = 5%, (**a-ii**,**b-ii**) x = 10%, (**a-iii**,**b-iii**) x = 15%, and (**a-iv**,**b-iv**) x = 20% (*w* = 12 nm, *h* = 6 nm).

Figure 6 depicts the electron probability density in the Γ-CB for both the ground and first excited states for the Ge/GeSn/Ge QD systems containing different amounts of Sn. The high probability in the Γ-CB ground state provides clear evidence that the electrons are confined within the GeSn QDs. However, the significant wave function indicates that leakage occurs into the Ge substrate and the cap layer owing to the limited band offset. In contrast, in the first excited state, the electron probability is low (~0.5–0.6) when the Sn content is low because of the smaller CB band offset energy and the small effective masses of electrons. Upon increasing the Sn content, the increased Γ-CB band offset energy increases the degree of electron confinement in the first excited state within the GeSn QDs, thus increasing the electron probability in the first excited state in the Γ-CB.

**Figure 7:**
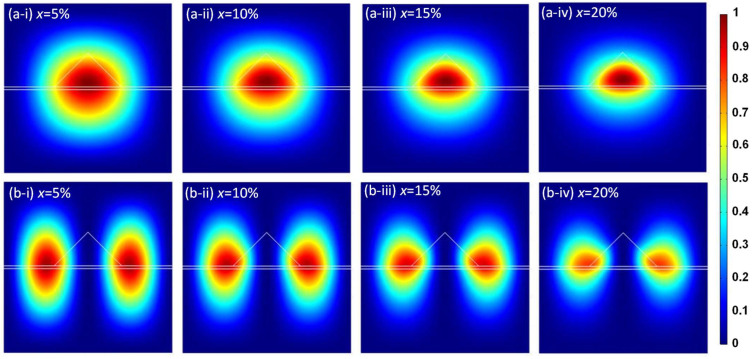
Heavy hole probability densities for the Ge/Ge_1-x_Sn_x_/Ge QD system in (**a**) the ground state and (**b**) the first excited state for the following Sn contents: (**a-i**,**b-i**) x = 5%, (**a-ii**,**b-ii**) x = 10%, (**a-iii**,**b-iii**) x = 15%, and (**a-iv**,**b-iv**) x = 20% (*w* = 12 nm, *h* = 6 nm).

Furthermore, Figure 7 shows the hole probability densities for the ground and first excited states in the HH bands of the Ge/GeSn/Ge systems with different Sn contents. Evidently, at a lower Sn content, hole confinement is limited owing to the smaller energy-band offset. Therefore, a certain hole probability density appears to exist in the Ge cap layer and substrate. Upon increasing the Sn content, the HH energy band offset increases from 0.1 eV (x = 5%) to 0.35 eV (x = 20%), significantly increasing the hole probability in the HH band.

**Figure 8:**
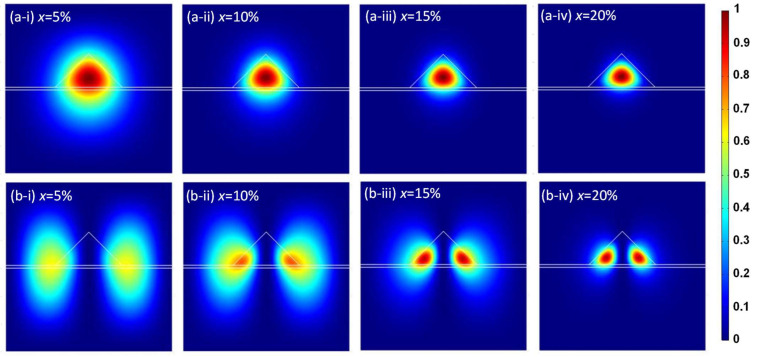
LH probability densities for the Ge/Ge_1-x_Sn_x_/Ge QD system in (**a**) the ground state and (**b**) the first excited state with the following Sn contents: (**a-i**,**b-i**) x = 5%, (**a-ii**,**b-ii**) x = 10%, (**a-iii**,**b-iii**) x = 15%, and (**a-iv**,**b-iv**) x = 20% (*w* = 12 nm, *h* = 6 nm).

The hole probability densities in the LH band for the ground and first excited states of the Ge/GeSn/Ge systems containing different Sn contents are shown in Figure 8. This figure demonstrates that at lower Sn contents, the holes are not entirely confined inside the GeSn QDs because of the smaller energy band offset, similar to the case of the HH band. However, at higher Sn contents, the energy band offset is increased, as shown in Figure 3a–d. Consequently, the degree of hole confinement increases inside the GeSn QDs, thereby increasing the probability density of holes in the LH bands.

**Figure 9:**
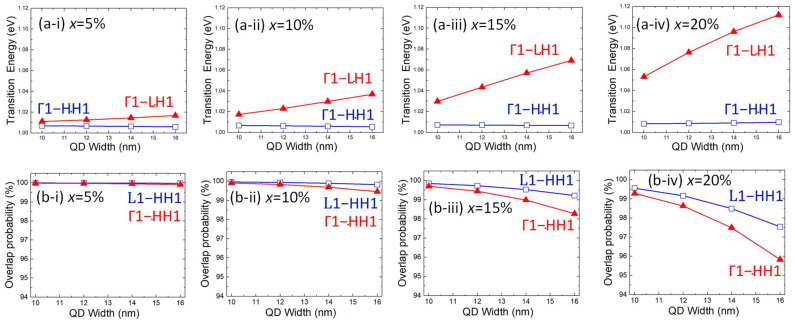
(**a**) Transition energies and (**b**) electron–hole overlapping wave function probabilities in the Ge_1−x_Sn_x_ QDs on a Ge substrate for the following Sn contents: (**a-i**,**b-i**) x = 5%, (**a-ii**,**b-ii**) x = 10%, (**a-iii**,**b-iii**) x = 15%, and (**a-iv**,**b-iv**) x = 20%.

Figure 9a shows the direct and indirect bandgap transition energies as a function of the QD width for the Ge/GeSn/Ge QDs with different Sn contents. Although the Γ-CB lies lower than the L-CB at higher Sn contents, the energy states of Γ-CB sit above those of the L-CB. These results indicate that a higher energy is required to raise an electron from HH1 to Γ1 than to raise an electron from HH1 to L1. Thus, the Γ1 → HH1 transition requires a higher energy than L1 → HH1. Meanwhile, the probability of overlap in the electron–hole wave functions is plotted as a function of the QD width in Figure 9b, which shows that a greater width shifts the electron and hole wave functions further from one another. Consequently, the probability of an electron–hole wave function overlap decreases with the increasing QD width. However, note that for a Sn content of 20%, the Ge/GeSn/Ge system exhibited an electron–hole overlap probability of >96%, even for a 16 nm thick QD. The localisation of photogenerated carriers improves the optical responsivity [44], enhancing the sensing capacity. Furthermore, the good overlap probability gives clear evidence of high carrier confinement, which decreases scattering and recombination losses, including SRH, radiative, and Auger losses, thereby reducing the dark current. Therefore, the QD-based PDs are feasible candidates for optical detection, even at high temperatures [24,25,26,27,28,29].

**Figure 10:**
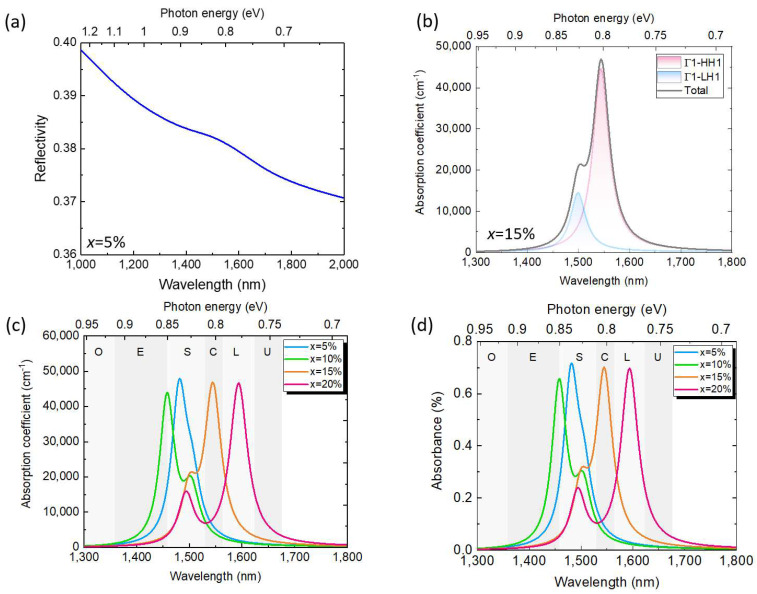
(**a**) Simulated reflectivity of the Ge_0.95_Sn _0.05_ QD on a Ge substrate. (**b**) Simulated TE−polarized absorption coefficient spectra for Γ1–HH1 and Γ1–LH1 transitions and the superposition of their Ge_0.85_Sn_0.15_ QD on a Ge substrate. (**c**) Simulated TE−polarized absorption coefficient spectrum and (**d**) absorbance spectrum of the Ge_1−x_Sn_x_ QDs on a Ge substrate with different Sn contents (*w* = 12 nm, *h* = 6 nm).

For photodetectors, the reflectivity and optical absorption coefficient are important parameters impacting the sensing performance. We therefore investigated the reflectivity and optical absorption coefficient spectra of the Ge/GeSn/Ge QDs with different Sn contents and *w* = 12 nm and *h* = 6 nm. The reflectivity (R) and transmittance (T) spectra were calculated through an FEM simulation, and the refractive indices of the materials were taken from Ref. [45]. A plane wave was used as the light source, which was normally incident onto the Ge/GeSn/Ge QD structure in air. The absorbance of the Ge/GeSn/Ge QD was then obtained using A = 1−T−R. Figure 10a shows the simulated reflectivity spectrum of the Ge_0.95_Sn_0.05_ QD on a Ge substrate. The reflectivity is ~40% at λ = 1000 nm and then decreases with the increasing wavelength owing to the decreased refractive index of the materials. The reflectivity is dominated by the reflection at the interface between the Ge cap layer and air, because the dimensions of the GeSn QD are much smaller than the wavelength. The Ge/GeSn/Ge QDs with other Sn contents exhibit similar results. Figure 10b shows the calculated TE-polarised optical absorption coefficient spectra for the Γ1–HH1 and Γ1–LH1 transitions, as well as their superposition for the Ge/GeSn/Ge QDs with x = 15%. The Γ1–HH1 transition occurs at a lower energy and yields a larger absorption coefficient than the Γ1–LH owing to the larger squared momentum matrix. Thus, a high absorption coefficient of >10,000 cm^−1^ can be achieved for efficient optical absorption. Figure 10c shows the optical absorption spectra for the Γ1–HH1 and Γ1–LH1 transitions in their Ge/GeSn/Ge QDs with different Sn contents and *w* = 12 nm and *h* = 6 nm. As the Sn content changes, the bandgap energy also varies, thus shifting the absorption coefficient spectra. The simulated absorption coefficient spectra can cover the telecommunication S, C, and L bands with high optical absorption coefficients. Figure 10d shows the calculated absorbance spectra for one layer of the GeSn QD. Despite the thin thickness of the GeSn QD, an absorbance of ~0.5% can be achieved. The absorbance can be significantly enhanced by adopting a multi-stacked QD structure to increase the thickness of the active layer, thereby yielding more photocurrent. These results indicate that the proposed Ge/GeSn/Ge QDs are promising materials for photodetectors that can operate in telecommunication bands for a wide range of applications.

## 4. Conclusions

A theoretical study of the type–I GeSn QD structure was carried out using QDs on a Ge substrate with a Ge cap layer. The Sn content in the GeSn alloy was also varied (i.e., 5–20%) to determine its effect on the investigated properties. A lower energy band offset led to poor carrier confinement, which in turn decreased the carrier probability, especially in the higher-energy states. Interestingly, this problem was circumvented by increasing the Sn content in the QDs. More specifically, at higher Sn contents, the GeSn active layer possessed a direct bandgap, which supports a recently introduced telecommunication window. Furthermore, owing to the quantization of the energy states, a high carrier probability and a high electron–hole overlap were attained, rendering this system suitable for use in high detection operations at lower biases. On the other hand, owing to the lower scattering effect, the proposed QD detector can achieve a low dark current, thereby increasing the sensitivity. Overall, it can be concluded that GeSn QDs supported on a Ge platform are very promising materials for use in optoelectronic and sensing applications.

## Figures and Tables

**Table 1:** Bandgap energy parameters of Ge, and Sn.

	$E_{V,av}$ (eV)	∆ (eV)	$E_{g}^{\Gamma }$ (eV)	$E_{g}^{L}$ (eV)
Ge	0 [39]	0.29 [39]	0.7985 [8]	0.664 [8]
Sn	0.69 [39]	0.8 [39]	−0.413 [8]	0.092 [8]

## Data Availability

The data presented in this study are available upon request from the corresponding author. The data are not publicly available due to a commercial privacy policy.
